# Navigating the integration of knowledge and research evidence in clinical practice for children's foot health: A multi‐professional survey

**DOI:** 10.1002/jfa2.12034

**Published:** 2024-07-24

**Authors:** Lisa Hodgson, Carina Price, Julie Reay, Chris Nester, Stewart C. Morrison

**Affiliations:** ^1^ School of Sport and Health Sciences University of Brighton Eastbourne UK; ^2^ School of Health and Society University of Salford Salford UK; ^3^ School of Health Professions Keele University Keele UK; ^4^ School of Life Sciences & Medicine King's College London London UK

**Keywords:** allied health professional, evidence based practice, paediatric

## Abstract

**Background:**

Access to clinical services for children with foot and ankle problems are important, but unravelling the complexity of practice and service delivery can be challenging. The pursuit and implementation of research evidence is critical for driving positive change in practice, but little is understood about the approaches to knowledge and research acquisition in children foot health.

**Aim:**

The aim of the study was to: (1) explore multi‐professional habits of knowledge and research evidence acquisition in children's foot health; and (2) understand how clinicians integrate information for children and their families into their practice.

**Methods:**

This was a descriptive, cross‐sectional online survey. Participants were included if they worked in the UK and had experience of working within paediatric services.

**Results:**

There were complete responses from 247 health professionals, representing physiotherapists (*n* = 160), podiatrists (*n* = 50), orthotists (*n* = 25), nurses and specialists in community public health nurses (health visitors) (*n* = 12). Three main themes were generated from the data: (1) Factors that influence knowledge and inform clinical practice. (2) The role of Professional Bodies in informing professional knowledge. (3) Health Professionals' views on managing health information for parents and caregivers.

**Conclusions:**

This work advances understanding of the value health professionals' place in the development of materials for informing professional knowledge, as well as highlighting some of the challenges with translation of knowledge into clinical practice. The findings offer a national perspective of health professionals working on children's foot health and have highlighted that some of the most valued influences on clinical practice come from peer‐to‐peer networking.

## 1 INTRODUCTION

The role of research evidence in shaping, enhancing, and driving positive change in clinical practice is unequivocal, but balancing research against individual values and preferences can be challenging [1]. Clinicians face barriers to delivering evidence‐informed care, and factors such as lack of time, limited access to research and inadequate research appraisal skills [2] are cited. Linked with this, clinicians have reported difficulty with keeping up to date with online information [3] and the shift in health information delivery and access adds complexity to clinical encounters and impacts on trust and confidence with clinical decision‐making [4].

Whilst there is a requirement for clinicians to be evidence‐informed and have skills in acquiring, appraising, and applying research evidence to inform their own practice [5], there is also a growing expectation to navigate and appraise publicly available online material [6]. Whilst the benefits of accessible health information via the Internet are clear, this has also created new challenges for clinicians, particularly where the quality of health information is unknown [7]. Online forums are commonly used by parents and caregivers seeking health information [8, 9, 10] and offer an unfiltered perspective of healthcare experiences and may capture discussion that parents/caregivers feel are lacking in interactions with professionals. A related concern for health professionals is the extent to which online health information can influence health behaviours [11]. This is particularly notable in musculoskeletal development where parents/caregivers often raise concerns about their child's development, such as their walking or running and foot position [12, 13] and creates additional demand on health services [14]. A previous study of allied health professionals working with children experiencing foot and ankle problems [15] identified that clinicians struggled to find credible resources to support their practice, and this impacted on how they engaged with parents and caregivers during consultations. This work highlighted the need for future research on the habits of evidence acquisition, application, and sharing with service users.

Unravelling the complexity of footcare needs and promoting the appropriate services for children is challenging. A lack of coherence when developing services has been reported [16], and many components of practice lack robust, evidence‐based recommendations [13, 17, 18]. This is challenging for health services as a lack of understanding can lead to inappropriate referrals, which impacts health resources through generating greater demand and expectation. As such, it is important to understand more about the landscape of health professionals working in children's foot health, and the objective of this study was to explore multidisciplinary habits of knowledge and research evidence acquisition in children's foot health. The secondary objective was to understand how clinicians navigate and integrate health information into their practice.

## 2 METHODS

### 2.1 Study design

This was a descriptive, cross‐sectional online survey of health professionals practicing in the UK. The survey was conducted via the Online Survey (onlinesurvey.ac.uk) platform, a General Data Protection Regulation compliant tool for creating online surveys, and is reported in accordance with Checklist for Reporting Results of Internet E‐Surveys [19].

### 2.2 Survey development

This design of the survey instrument was informed by previous qualitative work [20]. The survey consisted of five sections: (1) demographics, (2) professional practice, (3) professional information about children's foot health, (4) views and experiences of foot health information and (5) the role of health professionals. The overall survey consisted of 28 questions across the sections and were a mix of open‐ (9) and close‐ended questions (19). Close‐ended questions consisted of both multiple choice and dichotomous questions and there were nine ‘other, please specify’ questions used to expand on closed questions. Open‐ended questions used free‐text boxes, which allowed respondents to answer without any restrictions. Open‐ended questions were used to provide explanation and develop narrative beyond the scope of the closed questions [21]. They explored practitioners' views and opinions about behaviours adopted when informing professional knowledge and to identify barriers to the acquisition and application of knowledge in clinical settings.

Prior to launching the survey, we conducted a pilot study where we tested the survey questions, length and completion time. Four colleagues with professional expertise ranging from health psychology, social science and sport science backgrounds were invited to participate in this pilot testing. Feedback was provided in a 20–30‐min cognitive debriefing interview where the researcher (LH) obtained information about participant's experiences of using and navigating the survey. Using a debriefing exercise provided an opportunity to evaluate and develop understanding of the effectiveness and interpretation of survey questions, structure and format to reduce response error [22, 23]. The survey was then edited and launched and was live for 5 months (November 2018–March 2019). Ethical approval for this work was granted by the School of Health Sciences Research Ethics Panel, University of Brighton.

### 2.3 Participant recruitment

A purposeful sampling approach was adopted. Participants were included if they worked in the UK and had recognised professional accreditation and experience of working within paediatric services, for example, this included several work settings, environments and professional backgrounds (Health visitors, physiotherapists, podiatrists, GPs, midwives, nurses, etc.). The survey was promoted using social media to optimise geographical reach and to easily share the survey among a professional population and increase responses [24, 25]. In addition, the research team contacted health professional organisations such as those representing paediatric physiotherapists, podiatrists and nurses who distributed the survey link via social media accounts and electronic newsletters.

Participants were able to review information about the study on a participant information page (the landing page) before proceeding to providing electronic consent, which was mandatory prior to participation. The landing page included information about the purpose of the study, inclusion/exclusion criteria, completion of the survey and data management. The online survey platform enabled the researchers to collate responses anonymously ensuring that data/responses could not be identified or linked back to an individual. No personal data were recorded.

### 2.4 Data analysis

All data were exported to Excel© (Microsoft) for cleaning and analysis. The lead author cleaned the data, removed responses from non‐UK practitioners (5) and, incomplete entries (9). The survey generated qualitative and quantitative responses. Descriptive statistics were used to summarise quantitative close‐ended questions and were analysed by the lead author (LH). Two authors (LH and JR) analysed the qualitative responses using summative content analysis [26]. Key words and phrases were coded, counted for frequency and grouped into main themes. This enabled the researchers to obtain meaning and context directly from the text information provided in the survey [26]. Preliminary themes were shared, discussed, modified and finalised with the research team to form the final themes presented in this paper.

## 3 RESULTS

### 3.1 Demographics, conditions and advice

Data collection was undertaken via an online survey and there was no single response rate. We had a view response of 697, and 261 complete responses. After the data were cleaned, there 247 remained (207 female, 37 males, 3 did not declare). The largest regional response was from the Southeast of England (33% of responses) followed by the North East (18%), North West (10%), and the Midlands (10%). The largest professional response was from physiotherapists (61%), followed by podiatrists (19%), orthotists (10%), nurses and specialist community public health nurses (health visitors) (5%). Most participants (58%) had >15 years' professional experience, whilst 17% had 10–14 years of experience. Health professionals worked predominantly in the National Health Service (*n* = 199) or private practice (*n* = 48). Participants worked with a breadth of children within the clinical setting and this spanned neurological and neuromuscular conditions, systemic disease, orthopaedics and developmental concerns. The most common foot problem encountered by health professionals was pes planus. The three most common areas where health professionals provided advice to parents and caregivers were: lower limb development (e.g., in‐toeing) (reported by 74% of respondents), footwear/fitting, (reported by 72% of respondents) and the development of children's feet (reported by 58% of respondents).

Three main themes were generated from the data. (1) Factors that influence knowledge and inform clinical practice. (2) The role of professional bodies in informing professional knowledge. (3) Health professionals' views on managing foot health information for parents/caregivers.

### 3.2 Factors that influence knowledge and inform clinical practice

Health professionals adopted mixed approaches to informing their professional knowledge, and this included journals (for which 78% of respondents rated as useful or extremely useful), professional networks (90%), textbooks (57%), and (professional) online forums (65%). In contrast, 48% of respondents were ‘*unsure*’ if forums for parents and caregivers were useful and 91 clinicians considered them unhelpful (28%) or extremely unhelpful (9%). Fifty‐one percent of respondents (*n* = 132) reported that discussion with professional colleagues was an extremely useful resource for informing their professional practice: *‘when requiring information or knowledge, consulting with or engaging with colleagues could be most beneficial for developing a diagnosis or a course of treatment’; ‘the exchange of clinical experience can be the best and direct resource a health professional can engage with’*.

Respondents (91%) indicated that professional groups or seeking professional colleagues' advice and knowledge was influential in clinical decision‐making *and ‘contributed to professional development’*. When asked how it shaped clinical thinking, most respondents (85%) reported value in discussing cases with colleagues and that this helped develop ‘*critical thinking*’ and provided ‘*opportunity to exchange professional viewpoints*’ and ‘*apply latest evidence base*’ (89%).

### 3.3 Role of professional bodies in informing professional knowledge

Thirty‐four percent of respondents reported that their professional body did not share or promote relevant research and 35% were unsure. Physiotherapists (14% of total respondents) and orthotists (15% of total respondents) were the most common professions to report that their professional body shared research evidence with its members.

Survey respondents were asked to describe the types of information they received from their Professional Body and responses included news bulletins, newsletters, leaflets/information sheet for families, and information signposting to research. Social platforms were used to share relevant research papers, to link in with training events and conferences.

Information provided by professional bodies was reported to be good (*n* = 55/247, 22%) or excellent (*n* = 22/247, 9%), but 60% of survey respondents did not answer this question. Thirty‐four percent of the survey participants (*n* = 90) believed that professional bodies could do more to develop, share and inform members' knowledge about children's foot health research and practice. More than half of the professionals (57%) used information produced by other professions. Of those not using information from other professional bodies, it was highlighted that they did not use information as they did not have close working networks with other health professionals; *‘[do not] access any information‐we do not work closely with orthotists or podiatry unfortunately’* or information was seen to lack relevance, ‘… [*it is*] *too general. The information produced at our children's hospital is more targeted and from the MDT [multi‐disciplinary team] so more rounded rather than being from one profession. Too many that I see focus on the foot position without referencing the range of normal and the function of the foot*’.

### 3.4 Health professionals' views on managing available foot health information for parents and caregivers

Engaging with parent‐facing information on the Internet was common for 50% of the health professionals (*n* = 130). When considering the quality of these resources, most professionals reported web‐based material found on the NHS, professional body sites and general online health websites was ‘*good*’. Less than one in five (18%) rated these sites as ‘Excellent’ resources for parents and caregivers. Despite this, a considerable proportion of respondents reported that there was no consistency with children's foot health messages on the Internet (*n* = 116, 44%) or that they were unsure (*n* = 110, 42%). Health professionals noted that conflicting messages arose―foot health information was *‘unclear’,* there were *‘different approaches to foot health’,* it was *‘under researched’* and subject to ‘*conflicting advice’.*

Over half of the respondents (*n* = 154 from 247, 64%) believed that health professionals had a role in both providing good foot health information and managing the quality of information that parents and carers can access. Although noting that it was difficult to challenge web‐based content from an online forum, many health professionals highlighted that web‐based content often was discussed in consultations, and this was seen as an opportunity to challenge *‘the misconception of acquired knowledge’* or explain the information in‐depth to improve understanding, accuracy and awareness of foot health information. Health professionals believed that education was important as reported by one health professional: ‘*It is very difficult to police what is on the Internet so making sure parents have a good understanding of what is good quality information is the way forward’,* ‘ensuring it is evidence based’ was important. Another respondent noted that ‘*As health professionals we have little effect on the information that exists, independent factual information needs to be presented to new parents in an easily accessible format*’.

Many health professionals have used information about children's foot health produced by other professional bodies (*n* = 141, 57% of the respondents). Physiotherapists (*n* = 103 of 134; 77% of physiotherapists who responded) and podiatrists (*n* = 31 of 56; 55% of podiatrists who responded) indicated that they used booklets or web‐based material for parents and caregivers to access. Twenty‐one orthotists (53% of the 40 who replied) indicated that they were unsure.

## 4 DISCUSSION

This study represents a synthesis of how health professionals working in children's foot and ankle services inform their professional knowledge and clinical practice. It advances understanding of the value health professionals place on the development of materials for informing professional knowledge, as well as highlighting some of the challenges with translation of knowledge into clinical practice. The findings from this survey build upon previous research into children's foot health, which highlighted some of the challenges with delivering evidence‐based practice, particularly when balancing against the demands of services [27]. Our previous work identified that continued professional development activities could be challenging and that many factors impacted the translation of knowledge into clinical delivery. The findings from this survey help to contextualise the factors that inform professional knowledge and how this is shaped for parents and caregivers.

Whilst there is a requirement for clinicians to be skilled in the acquisition, appraisal and application of research evidence to inform their practice [5], they also need to successfully navigate and appraise publicly available (online) materials [6]. Acquiring effective critical appraisal skills can be dependent on different factors and research suggests that formal critical appraisal teaching can improve knowledge, but the effectiveness of these skills can be dependent on individuals understanding of research [28]. Health professionals developed their professional knowledge by engaging with colleagues and valued the opportunity to apply critical and reflective thinking, which was perceived to enhance decision‐making [29]. Our findings extend earlier research [20, 27], which demonstrated that much of the evidence‐based literature on children's foot health lacked consistency. There were concerns with the currency of the information that is available and this raised questions of whether more needs to be understood about the evidence and barriers to children's foot health. Peer‐to‐peer and multi‐ and/or cross‐professional networks were being used to advance professional development, discuss literature and apply them to clinical practice. Our data identified that professional networks were perceived as more useful than journals (101 v 62 respondents rated as extremely useful) and there were many reasons to explain this finding, particularly as some clinicians struggled with research appraisal [28]. Professional bodies encourage members to engage with and demonstrate critical reflection as part of continual development to enhance practice, and evidence how new knowledge has added to their practice and skills [29]. Yet, our data indicated that the promotion of evidence‐based practice by professional bodies was inconsistent and differed across the professions. It also highlighted that there were inconsistencies with health professionals' awareness of materials provided by professional bodies.

The findings demonstrated that health professionals understood the importance of knowledge translation in practice and their role in supporting parents and caregivers' literacy. By doing so, it is recognised that information could improve caregivers' decision‐making and access to foot health services. Linked with this, our study has identified the common topics that health professionals discuss with parents and caregivers (e.g., lower limb development, children's footwear/fitting and development of the foot) and highlighted some gaps in information they shared, such as information about footwear types and styles. This poses a challenge for health professionals as the quality of foot health information (often online) is poor, and knowledge often fails to filter through [30, 31]. Knowledge transfer, or implementation science, argues the importance of evidence in practice as stretching beyond the realms of continual professional development to create opportunities and include research evidence to inform all decision‐making groups [32]. Yet, insufficient pathways to knowledge affects the education and promotion of health messages to consumers (e.g., parents and caregivers, health professionals and other stakeholders). As such, efforts beyond typical scientific dissemination are important to ensure translation of evidence into practice [33, 34] and innovations are an important strategy for health professionals to consider.

The findings reported in this study offer a perspective of health professionals working in children's foot health in the UK and the findings represent a broad range of health professional disciplines. Our findings highlight the additional challenges of maintaining awareness of the resources and/or websites that parents and caregivers use for accessing health information, and the importance of promoting credible, evidence‐informed sources to support literacy and debunk misinformation. Linked with this, it is also important for health care professionals to embrace conversations with parents and caregivers about the information they are accessing. However, we acknowledge that the overall numbers representing the different professions in this study was small. We also acknowledge that our recruitment approach may have introduced sampling bias into the study, and the findings are specific to professionals based in the UK.

## 5 CONCLUSION

This study offers a UK‐specific perspective of health professionals working in children's foot health. The study demonstrated that some of the valued and most common influences on clinical practice come from peer‐to‐peer networking. Seventy percent of the clinicians reported that their professional body did not, or that they were not sure their professional body did, share or promote relevant health research. Similarly, awareness about types of information shared or produced for parents and caregivers about children's foot health appear low and differed between professions. The findings demonstrated that health professionals understood the importance of knowledge translation in practice and that areas where parents and caregivers needed guidance was related to footwear and fitting as well as foot development. In addition, parents and caregivers needed further information about these topics to expand understanding and approaches to supporting their children's foot health.

## AUTHOR CONTRIBUTIONS

**Lisa Hodgson**: Conceptualisation; methodology; project administration; data curation; formal analysis; writing – original draft; writing – review & editing. **Carina Price**: Methodology; data curation; formal analysis; writing – original draft; writing – review & editing. **Julie Reay**: Formal analysis; writing – original draft; writing – review & editing. **Chris Nester**: Funding acquisition; conceptualisation; methodology; supervision; writing – original draft; writing – review & editing. **Stewart C. Morrison**: Funding acquisition; conceptualisation; methodology; supervision; data curation; formal analysis; writing – original draft; writing – review & editing.

## CONFLICT OF INTEREST STATEMENT

Prof Chris Nester is Chair of the Scientific Committee of Great Foundations (Central and Northwest London NHS Foundation Trust Charitable Fund). Dr Stewart Morrison is the deputy editor of the Journal of Foot & Ankle Research. All other authors declare no conflict of interest.

## ETHICS STATEMENT

Ethical approval for this work was granted by the School of Health Sciences Research Ethics Panel, University of Brighton.

## PATIENT CONSENT STATEMENT

All participants provided consent for publication.

## Supporting information

Supporting Information S1

## Data Availability

The datasets used in this work are available from the corresponding author on reasonable request.
