# Supplementary material for: Navigating the integration of knowledge and research evidence in clinical practice for children's foot health: A multi‐professional survey
Source: J Foot Ankle Res. 2024 Jul 24;17(3):e12034. doi: 10.1002/jfa2.12034 (PMC11633346; doi:10.1002/jfa2.12034)
Supplement: Supplementary file 1 — Supporting Information S1 [file JFA2-17-e12034-s001.pdf]

What health professionals think about children's feet

Page 1: Participant Information & Consent

What do health professionals think about children's feet?

You have been invited to participate in an online survey that will explore health professional’s practices, knowledge, opinions and behaviours relating to children’s foot health. The aim is to identify mutual behaviours and opinions around how health professionals find and share information that is both relevant to health professional’s practice, as well as contribute towards parents understanding of children’s foot health.

Who is conducting the study: The study is being undertaken by Dr Stewart Morrison (Reader) and Ms Lisa Hodgson (Research Officer) at the University of Brighton. This study is also in collaboration with the University of Salford.

Participation: Participation is voluntary and you may exit from the survey at any time. If you wish to participate please continue to read this page and confirm your participation below.

Confidentiality: Information collected will only be assessable by the research team. The survey will not ask for contact details and therefore the research team won't hold your

contact details or will be able to identify participants.

Contact details: If you have any questions or queries about the survey please get in contact with the research officer Lisa Hodgson L.Hodgson@brighton.ac.uk

Thank you for taking the time to read the above information. Please remember the survey can be completed at any time. Should you decide not to participate today the survey link will be live for 3 months and available on our Great Foundations Website https://greatfoundations.org.uk/

Please select one of the options below. Clicking 'Agree' indicates that you have read and understood the above information and you voluntarily agree to participate. ✱ *Required*

- ◯ Agree
- ◯ Disagree

Page 2: Demographic Information

Basic demographic data will be collected. This information is only to indicate where the survey is reaching and for category purposes and it will not be used to identify participants.

Please select your gender:-

If you selected Other, please specify:

Please select the highest educational qualifications that you hold:-

If you selected Other, please specify:

Please select the region you live in:-

If you selected Other, please specify:

Page 3: Basic demographic information about your professional background

The following is requesting you to share information about your area of work and basic information about the types of patients you see. The questions will not ask you to share any personal detail about your patients.

Please indicate your profession:-

[ ]

If you selected Other, please specify:

[ ]

Please indicate whether you are working in:- (multiple responses can be selected)

- ☐ Private practice
- ☐ Academia
- ☐ NHS setting
- ☐ Industry
- ☐ Other

If you selected Other, please specify:

[ ]

How many years have you been qualified?

Thinking about your caseload, what percentage of your caseload is paediatrics? (please select a response that best represents the % reflective of your current workload)

In your professional capacity do you believe you have a role in providing parents with children's foot health information?

If no, please indicate who you feel should provide this information to parents?

Please describe the common foot problems you encounter in practice. What causes these issues?

Page 4: Searching for children's foot health information

The next section will explore how health professionals currently search and find information about children’s foot health.

When you search for research material on children's feet; how useful do you find the following resources?

Please don't select more than 1 answer(s) per row.

	Extemely useful	Useful	Not sure	Unhelpful	Extremely unhelpful
Online journals	☐	☐	☐	☐	☐
Professional related networks (e.g. any professional network/special interest groups you are a member of)	☐	☐	☐	☐	☐
Other health professional colleagues	☐	☐	☐	☐	☐
Textbooks	☐	☐	☐	☐	☐
Professional online forums	☐	☐	☐	☐	☐
Parent online forums	☐	☐	☐	☐	☐

Are there any other resources that you access? (e.g. online resources, groups or social media)

Which of the above rescources commonly influence your clinical decision making?

How do these resources influence the clinical thinking process?

Does your professional body provide you with information or updates on the latest research into children's foot health that can be used to inform your own knowledge and clinical practice?

If yes, please indicate what types of information that you have received?

In your view how do you rate the relevance of the information provided by your professional body?

Please don't select more than 1 answer(s) per row.

	Excellent	Good	Not sure	Poor	Very poor
Select appropriate response	☐	☐	☐	☐	☐

Do you feel that your professional body does enough to inform and develop your own knowledge of children's foot health?

Have you ever used information about children's foot health produced by another health professional area/body?

If no, Why did you choose not to use this information?

If yes, can you please indicate what type of information this was? (e.g content, types of information, format of information etc)

In your view, what areas of children's foot health do you feel research should focus on to advance knowledge and clinical practice?

Page 5: Parent facing information

Our research is telling us that parents are using the internet to access a number of online resources to inform their knowledge and understanding of a number of health conditions. This section explores health professional's views and opinions on current foot health messages that are conveyed to parents using general online health or parents facing forums /websites.

Have you accessed online resources for parents on children's foot health?

Our research indicated that parents are using the internet and different websites to access health information, can you tell us how you would rate the quality of these online resources?

Please don't select more than 1 answer(s) per row.

	Excellent	Good	Not sure	Poor	Very poor
NHS websites	☐	☐	☐	☐	☐
Professional body websites	☐	☐	☐	☐	☐
Footwear companies websites	☐	☐	☐	☐	☐
Online parent forums (e.g. Net mums, Mumsnet, Babycentre)	☐	☐	☐	☐	☐
Online magazines (e.g. Parent, Mother & Baby)	☐	☐	☐	☐	☐

Other health websites (e.g. WebMD)	☐	☐	☐	☐	☐
Non-health/ commercial websites (e.g.blogs)	☐	☐	☐	☐	☐

Are there any other online resources you use and is not captured in the above list?

Do you think there are consistent children's foot health messages on the internet?

If no, please tell us why you believe they are not consistent?

How do you believe is best to manage inconsistent information online? (This could be based on some of your own clinical practice experiences, engagement with parents, or

based on your own experiences of searching for information etc)

Do you believe health professionals have a role in managing the quality of foot health information found on the internet?

Does your professional body produce material about children's foot health for parents? (e.g. leaflets, booklets that they can take away with them, digital material etc)

If you selected Other, please specify:

If yes, please tell us what format this information is presented in? (multiple answers can be selected)

- ☐ Leaflets
- ☐ Websites
- ☐ Booklets
- ☐ Other

If you selected Other, please specify:

Please tell us what types of information they provide parents? (e.g. advice, information about foot health and conditions, development of the foot etc)

What are the most common areas of advice you share with parents about children's foot health? **Please select top 3 common areas.**

- ☐ Information about footwear/fitting/types
- ☐ Information about milestone events
- ☐ Development of the foot
- ☐ Orthoses/In-soles
- ☐ Specific conditions (e.g. Skin conditions)
- ☐ Lower limb development (e.g. In-toeing)
- ☐ Foot specific issues (e.g. Talipes)
- ☐ Other

If you selected Other, please specify:

What information do you feel parents need to help them develop their own understanding of children's foot health? **(multiple answers can be selected)**

- ☐ General foot hygiene and maintenance
- ☐ How to prevent common foot health conditions (e.g. verruca's/Athletes foot)
- ☐ Advice about the use of developmental aids (e.g. Jumparoos, Bouncers, Walkers)
- ☐ Advice and information about developmental events (e.g. milestone information)
- ☐ Information about types of footwear (ideal styles) and measuring
- ☐ Information about when to buy footwear
- ☐ General information about how to support foot health in early childhood
- ☐ Information about specific health conditions
- ☐ Other

If you selected Other, please specify:

Please tell us how you feel is the best way to deliver relevant foot health messages to a parent audience?

Page 6: Final thoughts

Finally, is there anything else that you would like to tell or share with us about children's feet, your practice and/or concerns you hear from parents about children's foot health?

Page 7: Thank you

YOUR RESPONSES HAVE BEEN SUBMITTED

THE GREAT FOUNDATIONS RESEARCH TEAM WOULD LIKE TO THANK YOU FOR YOUR TIME AND COMMITMENT TO FILLING IN THIS SURVEY. YOUR CONTRIBUTION IS GREATLY VALUED.

For further information about the project or regular updates please visit our Website, Facebook and Twitter pages.

Website: https://greatfoundations.org.uk/
Twitter: https://twitter.com/GrtFoundations
Facebook: https://www.facebook.com/greatfoundationsfoothealth/

Key for selection options

2 - Please select your gender:-

Female
Male
I prefer not to answer

Other

3 - Please select the highest educational qualifications that you hold:-

Undergraduate (e.g BSc)
Postgraduate (e.g MSc)
Doctorate (e.g PhD)
Diploma
Other

4 - Please select the region you live in:-

South East England
South West England
North East England
North West England
Midlands
North Wales
South Wales
Scotland
Northern Ireland
Republic of Ireland
I prefer not to answer
Other

5 - Please indicate your profession:-

G.P
Health Visitor
Nurse
Orthotist
Physiotherapist
Podiatrist
Other

7 - How many years have you been qualified?

1-4 years
5-9 years
10-14 years
15 years or more

8 - Thinking about your caseload, what percentage of your caseload is paediatrics? (please select a response that best represents the % reflective of your current workload)

Less than 10%
10--19%
20-29%
30-39%
40-49%
50-59%
60-69%
70-79%
80-89%
90-100%

9 - In your professional capacity do you believe you have a role in providing parents with children's foot health information?

Yes
No
Not sure
I prefer not to answer

14 - Does your professional body provide you with information or updates on the latest research into children's foot health that can be used to inform your own knowledge and clinical practice?

Yes
No
Not sure

15 - Do you feel that your professional body does enough to inform and develop your own knowledge of children's foot health?

Yes
No
Not sure
I prefer not to answer

16 - Have you ever used information about children's foot health produced by another health professional area/body?

No

Yes
Not sure
I prefer not to answer
None available

18 - Have you accessed online resources for parents on children's foot health?

Yes
No

21 - Do you think there are consistent children's foot health messages on the internet?

No
Yes
Not sure

23 - Do you believe health professionals have a role in managing the quality of foot health information found on the internet?

Yes
No
Not sure

24 - Does your professional body produce material about children's foot health for parents? (e.g. leaflets, booklets that they can take away with them, digital material etc)

No
Yes
Not sure
Other
